# Supplementary material for: Complexity of cortical wave patterns of the wake mouse cortex
Source: Nat Commun. 2023 Mar 15;14:1434. doi: 10.1038/s41467-023-37088-6 (PMC10015011; doi:10.1038/s41467-023-37088-6)
Supplement: Supplementary file 1 — Supplementary Information [file 41467_2023_37088_MOESM1_ESM.pdf]

Supplementary information

Complexity of cortical wave patterns of the wake mouse cortex

Yuqi Liang, Junhao Liang, Chenchen Song, Mianxin Liu, Thomas Knöpfel, Pulin Gong and Changsong Zhou

Overview

Supplementary figures

Supplementary Fig 1	Determination of the local wave pattern detection threshold.
Supplementary Fig 2	Comparison of wave speeds calculated by phase velocity fields and amplitude in raw and filtered data.
Supplementary Fig 3	Curl and the divergence to show properties of the vector fields.
Supplementary Fig 4	Behavioral, physiological, and brain activity signatures of different brain states.
Supplementary Fig 5	Stability analysis
Supplementary Fig 6	Snapshot of wave patterns under awake (a) and anesthetized (b) states.
Supplementary Fig 7	Power spectrum density of wavenumber under awake and anesthetized states.
Supplementary Fig 8	The effects of one long-range connection on wave patterns in anesthetized and awake states.

Supplementary table

Supplementary Table 1	Regions with weak and strong long-range connection strength provided by Allen Mouse Brain atlas data.

1. Detecting complex waves in real and shuffled data

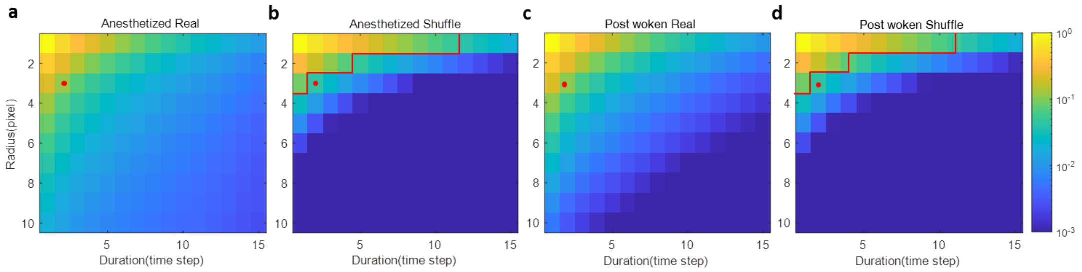

Supplementary Fig. 1: Determination of the local wave pattern detection threshold. The probability of local wave pattern detection is shown as a function of detection threshold (defined as a function of duration (d) and radius (r) threshold values). The colors represent the probability that a local wave pattern is detected in the actual data **a**,**c** and shuffled data **b**,**d** at both anesthetized and awake states. Shuffling was performed by randomization of the phase of the Fourier components of raw voltage signals, thus preserving the power spectrum on each pixel and random shuffling of pixel indices. The red line in the shuffled data is the 95% confidence for the unshuffled data to be due to spatiotemporally organized activity. The red dot indicates the wave detection threshold ($d = 2,\ r = 3$) used in the analysis.

2. Detecting complex waves in raw and filtered data

Supplementary Discussion: In order to assess the effects of phase analysis of the signal, we first identified – based on the PVF – the complex wave patterns then calculated their speeds based on amplitude gradients. The source patterns were detected and marked according to the PVF, as shown in **Supplementary Fig. 2a**, the bottom right part of the source pattern was the area of interest to calculate the speed because the pattern is centrosymmetric. There is less smoothing involved in amplitude gradients calculation than the phase vector field-based speed, because the latter is based on the Horn-Shunck optical flow method that introduces extra spatial smoothness and continuous constraints. More specifically, we used amplitude gradients to calculate the speed similar to Rubino et al which used phase[1]. Let A(x,y,t) be the amplitude of voltage activity at time t and coordinates x and y of the location. We calculated $\mathrm{speed(t)} = \left|\overline{(\partial \mathrm{A}/\ \partial \mathrm{t})}\right| / \overline{\|\nabla \mathrm{A}\|}$ for complex waves; **Supplementary Fig. 2 b** shows that the speed distributions are in the same order for the anesthesia trial in the raw and filtered data. We performed the same analysis on fully awake states; the result is shown in **Supplementary Fig. 2 c-d**. These analyses indicate that the effects of anesthesia and smoothing of phase analysis will not significantly affect the speed.

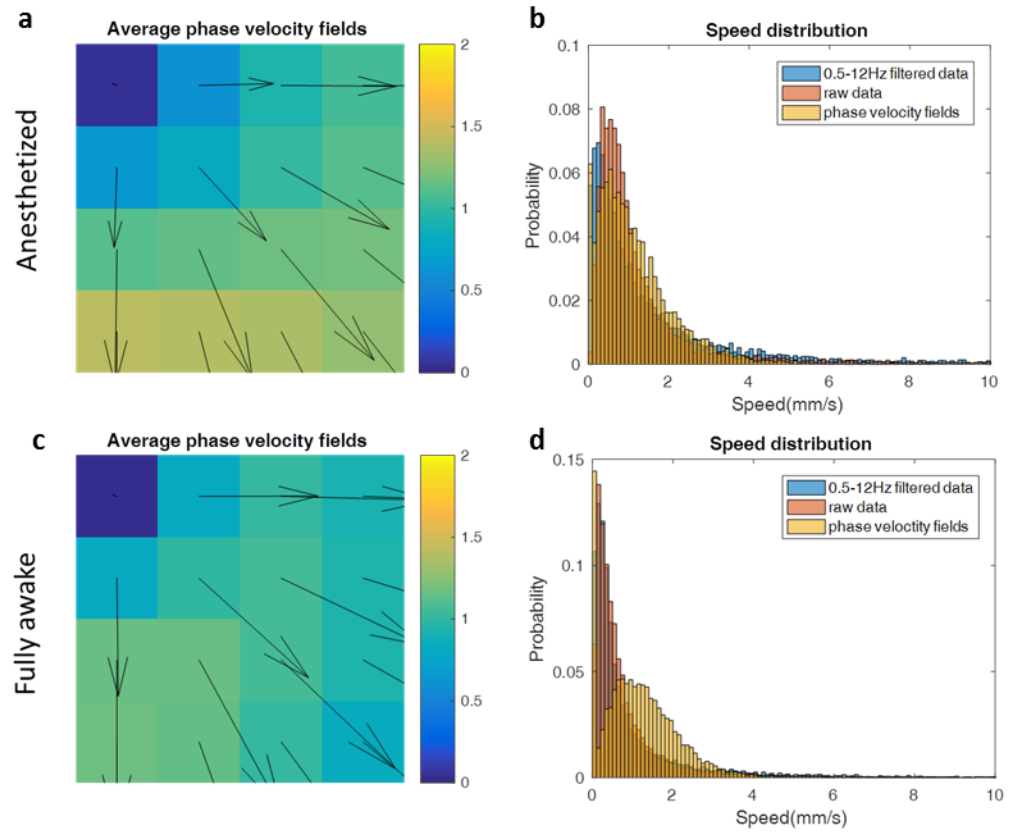

Supplementary Fig. 2: Comparison of wave speeds calculated by phase velocity fields and amplitude in raw and filtered data. a The bottom right portion of a source pattern averaged over 538 sources from mouse 1 anesthesia trial 2. Background color represents speed (mm/s). **b** Speed distributions of 538 sources from mouse 1 anesthesia trial 2. **c** The bottom right portion of a source pattern averaged over 704 sources from mouse 1 fully awake trial 2. Background color represents speed (mm/s). **d** Speed distributions of 704 sources from mouse 1 fully awake trial 2.

3. Curl and divergence on the PVF

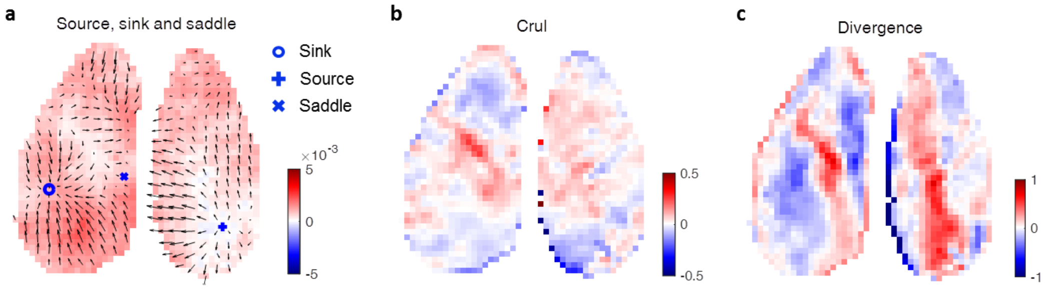

Supplementary Fig. 3: Curl and the divergence to show properties of the vector fields. a Phase velocity fields (PVFs) with singularities. Background color is the voltage amplitude. **b** Curl of the PVFs in (a). **c** Divergence of the PVFs in **a**.

4. Behavioral and brain features during recovery and fully awake states

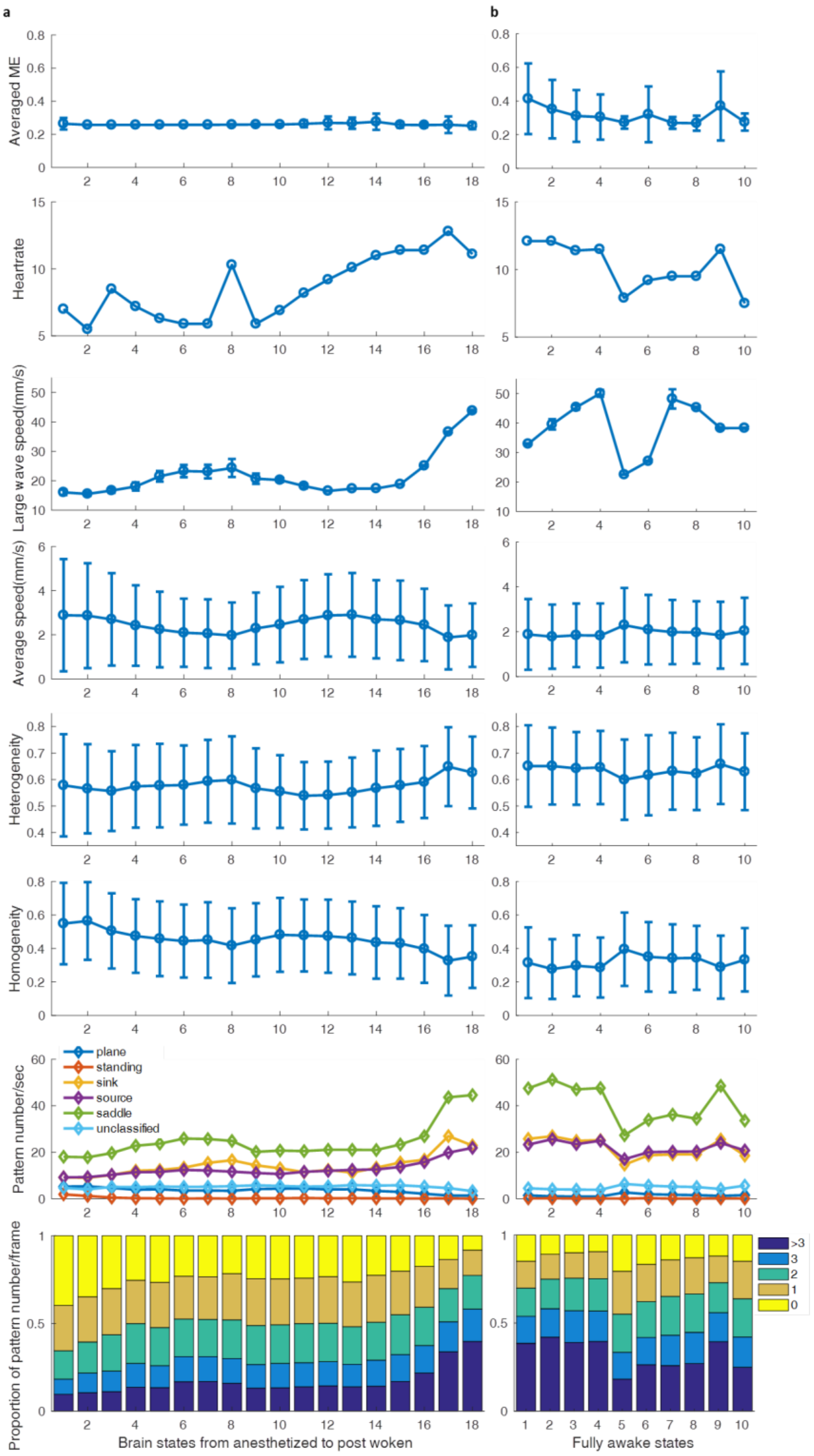

Supplementary Fig. 4: Behavioral, physiological, and brain activity signatures of different brain states on mouse 1, 18 trials from anesthetized to post woken and 10 trials for fully awake states. a During recovery from anesthesia, there is a smooth transition in mean motion energy, heart rate, and wave pattern properties. **b** Same as **a** for fully awake state. All the analysis shown is from the same mouse. Error bar presents the standard deviation of the quantity in time where applicable.

4. Further analysis of the neural field model

Homogeneous equilibria states

Supplementary Method: The homogeneous equilibria of Eq. (6-11) can be numerically obtained by solving the algebraic formulation after setting $a = 0, \frac{d}{dt} = 0, \nabla^2 = 0, \phi^{long-range} = 0$. This condition gives

$$Q_i = \frac{V_e - V_e^{rest} - g_e(V_e^{rest} - V_e)[(N_e^{cc} + N_e^{local})Q_e + I^{sc}]}{g_i(V_i^{rest} - V_e)N_i^{local}} \quad \text{(S1)}$$

$$Q_e = \frac{V_i - V_i^{rest} - g_i(V_i^{rest} - V_e)N_i^{local}Q_i}{g_e(V_e^{rest} - V_e)(N_e^{cc} + N_e^{local})} - \frac{I^{sc}}{N_e^{cc} + N_e^{local}} \quad \text{(S2)}$$

$$\Phi_e = (N_e^{cc} + N_e^{local})Q_e(V_e) + I^{sc} \quad \text{(S3)}$$

$$\Phi_i = N_i^{local}Q_i(V_i) \quad \text{(S4)}$$

$$\phi_e = Q_e(V_e) \quad \text{(S5)}$$

The state variables at equilibrium can be solved by a cross iteration of Eqs. S1 and S2. An important feature is that the model can display a 'bistable' region under certain parameter ranges (here stability refers to the stability without noise and spatial diffusion) (**Supplementary Fig. 5a**); complex spatiotemporal patterns can only emerge near the critical region of entering bistability when the anesthetic degree p decreases. Previous work of Steyn-Ross [2] showed that, when far away from the critical region, the homogeneous equilibrium will be very stable and requires a very large diffusion coefficient to generate spatial patterns. A very large diffusion coefficient of the gap junction is not biologically plausible due to the limited number of gap junctions present in the cortex, and therefore the diffusion coefficient is generally small, and a very stable homogeneous equilibrium will preclude pattern formation in such case.

Supplementary Discussion: A diagram illustrating the bistability of the system can be obtained using the anesthetic parameter p as the control parameter and calculating the equilibrium, which shows a fold bifurcation (**Supplementary Fig. 5a**). The middle branch (dashed line in **Supplementary Fig. 5a**) is unstable even without spatial diffusion. The bottom and upper branches are predicted to be stable without noise perturbations. We are interested in the case where $p = 0 \sim 0.5$. Thus, we take the lower branch as the reference equilibrium for our stability analysis, and the numerical simulation also starts with the equilibrium value in the lower branch.

When the system is poised around those equilibria and driven by noise, complex spatiotemporal patterns arise from breaking the symmetry, and the overall dynamic properties can be understood from the dispersion relation of the spatial stability analysis of the equilibria.

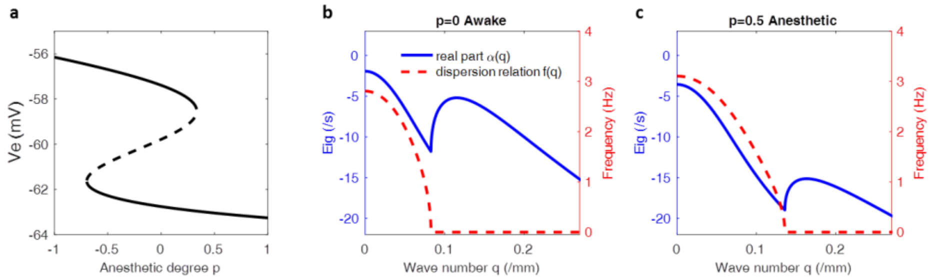

Supplementary Fig. 5: Stability analysis. a The homogeneous equilibrium value of V_e. In suitable range of p, multiple equilibria may exist. Solid/dashed black lines indicate the equilibrium is stable/unstable without spatial diffusion and noise. **b-c** The stability of the dispersion relations of the equilibria in the lower branch for $p = 0$ (**b**; simulating the awake state) and $p = 0.5$ (**c**; simulating the anaesthetized state). Red dashed line is the predicted dispersion relation $f(q)$, and blue solid line is the corresponding dominant eigenvalue real part $\alpha(q)$, with q being the wave number (spatial frequency). In the simulated anesthetized state **c**, the travelling waves dominate over Turing patterns while there is a strong competition between the travelling waves and Turing patterns in the case of wakefulness **b**.

Linear stability analysis

Supplementary Method: Linear perturbation theory can give insight into the local spatiotemporal stability of the equilibria in a scenario without noise and long-range effect (i.e. $= 0$,$\phi^{long-range} = 0$). It is convenient to transform the equations into first-order to perform the stability analysis. Let $F_e = \dot{\Phi}_e$, $F_i = \dot{\Phi}_i$, $f_e = \dot{\phi}_e$ and Eq. (6-11) become

$$\tau_e \dot{V}_e(x,y,t) = V_e^{rest} - V_e + (V_e^{rev} - V_e) g_e \Phi_e(t) + (V_i^{rev} - V_e) g_i \Phi_i(t) + D_e \nabla^2 V_e \quad \text{(S6)}$$
$$\tau_i \dot{V}_i(x,y,t) = V_i^{rest} - V_i + (V_e^{rev} - V_i) g_e \Phi_e(t) + (V_i^{rev} - V_i) g_i \Phi_i(t) + D_i \nabla^2 V_i \quad \text{(S7)}$$
$$\dot{\Phi}_e = F_e \quad \text{(S8)}$$
$$(\tau_d^E)^2 \dot{F}_e = -2\tau_d^E F_e - F_e + N_e^{cc}\phi_{eb} + N_e^{local} Q_e(V_e) + I_{sc} \quad \text{(S9)}$$
$$\dot{\Phi}_i = F_i \quad \text{(S10)}$$
$$(\tau_d^I)^2 \dot{F}_i = -2\tau_d^I F_i - F_i + N_i^{local} Q_i(V_i) \quad \text{(S11)}$$
$$\dot{\phi}_e = f_e \quad \text{(S12)}$$
$$r^2 \dot{f}_e = -2vrf_e - v^2\phi_e + v^2 Q_e(V_e) + v^2 r^2 \nabla^2 \phi_e \quad \text{(S13)}$$

Let $X = (V_e, V_i, \Phi_e, F_e, \Phi_i, F_i, \phi_e, f_e)$, Eqs. S6-S13 can be written in the form $\dot{X} = G(X) + D\nabla^2 X$. The homogeneous equilibrium X_0 is given by $G(X_0) = 0$ and its local spatiotemporal stability is predicted by the following Jacobian matrix. Consider a small perturbation $X(x,y,t) = X_0 + \delta X(x,y,t)$, where $\delta X(x,y,t)$ defined in $[0,L] \times [0,L] \times [0,\infty)$ can be linearly expanded in spatial Fourier series $\delta X(x,y,t) = \sum_{i,j\geq 0} A_{ij}(t)\, cos(\frac{\pi i x}{L} + \frac{\pi j y}{L})$. Denote $\vec{k} = (k_1, k_2) = (\frac{\pi i}{L}, \frac{\pi j}{L})$ as the wave number, it is sufficient to consider the stability of the k-mode perturbation, $\delta X_k(x,y,t) =$

$A_k(t)\, cos(\, k_1 x + k_2 y)$, separately. Linear approximation gives

$$\delta \dot{X}_k(x,y,t) = (\frac{\partial G}{\partial X}|_{X=X_0})\delta X_k(x,y,t) - D|k|^2 \delta X_k(x,y,t) = J(k)\delta X_k(x,y,t),$$

with the Jacobian matrix $J = (\frac{\partial G}{\partial X}|_{X=X_0} - D|k|^2) =$

$$\begin{bmatrix} -(1+D_e k^2 + g_e\Phi_e + g_i\Phi_i)/\tau_e & 0 & g_e(V_e^{rev}-V_e)/\tau_e & 0 & g_i(V_i^{rev}-V_e)/\tau_e & 0 & 0 & 0 \\ 0 & -(1+D_i k^2 + g_e\Phi_e + g_i\Phi_i)/\tau_i & g_e(V_e^{rev}-V_i)/\tau_i & 0 & g_i(V_i^{rev}-V_i)/\tau_i & 0 & 0 & 0 \\ 0 & 0 & 0 & 1 & 0 & 0 & 0 & 0 \\ N_e^{local}Q_e{}'(V_e)/(\tau_d^E)^2 & 0 & -1/(\tau_d^E)^2 & -2/\tau_d^E & 0 & 0 & N_e^{cc}/(\tau_d^E)^2 & 0 \\ 0 & 0 & 0 & 0 & 0 & 1 & 0 & 0 \\ 0 & N_i^{local}Q_i{}'(V_i)/(\tau_d^I)^2 & 0 & 0 & -1/(\tau_d^I)^2 & -2/(\tau_d^I) & 0 & 0 \\ 0 & 0 & 0 & 0 & 0 & 0 & 0 & 1 \\ v^2 Q_e{}'(V_e)/r^2 & 0 & 0 & 0 & 0 & 0 & -v^2k^2 - v^2/r^2 & -2v/r \end{bmatrix}$$

whose three of the 64 elements are k-dependent and $Q_b{}'(V_b) = \frac{\pi Q_b^{max} \exp\,(\pi(\theta_b - V_b)/(\sqrt{3}\sigma_b))}{\sqrt{3}\sigma_b(1+exp(\pi(\theta_b - V_b)/(\sqrt{3}\sigma_b)))^2}$. The dominant eigenvalue (with the largest real part) $\alpha(k) + i\omega(k)$ of J predicted that $\delta X_k(x,y,t) \sim e^{\alpha(k)}\cos(\omega(k)t + k_1 x + k_2 y)$. Since the parameters are independent of the positions (i.e. isotropic), the stability depends on $k = |\vec{k}|$ only. Three classes of activities can be classified: Hopf oscillation ($k = 0,\ \omega(k) \neq 0$), wave ($k > 0,\ \omega(k) \neq 0$) and Turing patterns ($k > 0,\ \omega(k) = 0$).

Note that the non-angular frequency and non-angular wave number are $f = \frac{\omega}{2\pi}$, $q = \frac{k}{2\pi}$. In the following, we use the non-angular forms f, q.

The stability of the equilibria in the lower branch of wakefulness ($p = 0$) and anesthetized states ($p = 0.5$) are shown in **Supplementary Figs. 5b, c** respectively. Modes with larger eigenvalue real parts $\alpha(q)$ are easier to be evoked by noise. We can see that in the case of the anesthetized state (**Supplementary Fig. 5c**), travelling waves ($f(q) > 0$) dominate over Turing patterns ($f(q) = 0$), hence the overall wave properties observed are more globally coherent (**Supplementary Fig. 6b**). On the contrary, in the wakeful state (**Supplementary Fig. 6a**), there is a strong competition between travelling waves ($f(q) > 0$) and Turing patterns ($f(q) = 0$), resulting in complex wave patterns [3].

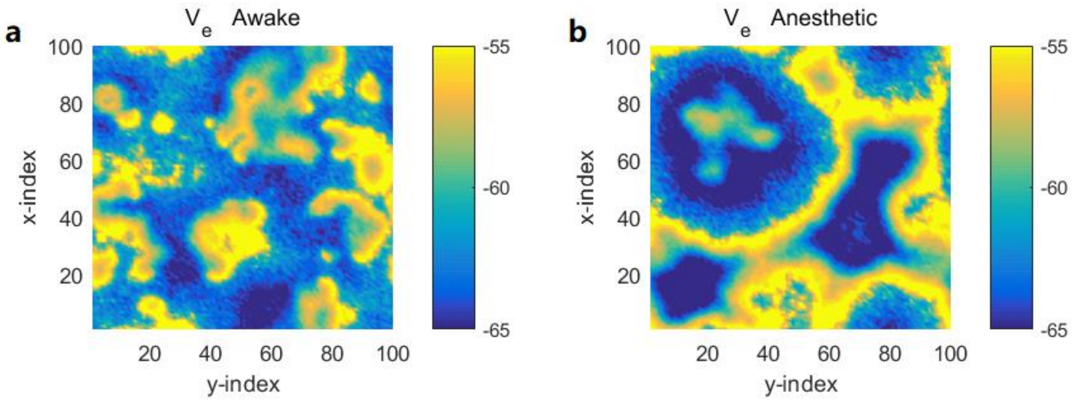

Supplementary Fig. 6: Snapshot of wave patterns under awake (a) and anesthetized (b) states. Colors represent the predicted population voltage in mV.

The difference in wavenumber or wavelength (**Supplementary Figs. 5b, c**)

between awake and anesthetic states can also be confirmed by numerical results. Each frame in the model simulation is a $L \times L = 100 \times 100$ image representing a 10mm × 10mm region (the spatial sampling frequency is 0.1 /mm). We can use two-dimensional spatial Fourier transform on the mean-detrended frame data. The normalized absolute value of the Fourier coefficients, $P(k_1, k_2)$, represents the power spectrum density at wavenumber (k_1, k_2), where k_1, k_2 ranges from -5/mm to 5/mm. Since the homogeneity of the neural field, $P(k_1, k_2)$ should take the same value for the same $k = \sqrt{k_1^2 + k_2^2}$ value and we can define a one-dimensional density $P(k) = sum\{P(k_1, k_2): k = \sqrt{k_1^2 + k_2^2}\}$. The normalized wavenumber density $P(k)$, with k ranging from 0.1/mm to 3/mm, for the anesthetic state (parameter $p = 0$, **main text Fig. 6a** blue) and the awake state (parameter $p = 0.5$, **main text Fig. 6a** bottom) are shown in **Supplementary Fig. 7**.

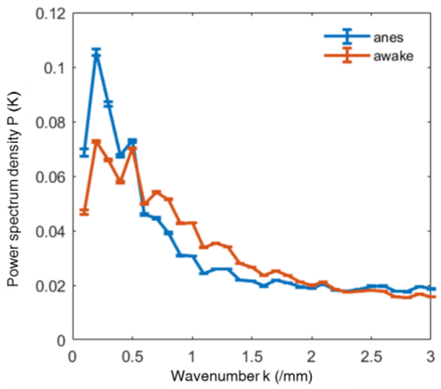

Supplementary Fig. 7: Power spectrum density of wavenumber under awake and anesthetized states. Error bars in the figure are SEMs for the simulation of 10 trials, where each trial has 6000 frames (60s duration with sampling frequency 0.1Hz).

Average wavenumbers $\langle k \rangle$ predicted from $P(k)$ in **Supplementary Fig. 7** are 1.08 /mm and 1.14/mm, resulting in average wavelengths ($\langle \lambda \rangle = 1/\langle k \rangle$) 0.93 mm and 0.88 mm, for the anesthetic and awake states, respectively. Thus, the more complex wave pattern in the awake state is accompanied with overall smaller average wavelength.

Wave speeds in the neural field model

Supplementary Method: The linear approximation gives the dispersion relation $q \sim f(q)$ and the corresponding stability degree $q \sim \alpha(q)$, as shown in **Supplementary Figs. 5b, c**. This relation cannot quantify the exact wave propagation velocity, but it can generate qualitative insight.

The dominant real part $\alpha(q)$ predicts the stability of mode q, which are all negative in our parameter setting. Thus, modes q with larger $\alpha(q)$ are more likely to emerge through destabilization from the homogeneous equilibria, and they emerge

with a temporal frequency $f(q)$. In our case, the dispersion relation $q \sim f(q)$ is not linear, so that the linear wave theory (which predicts the wave velocity as $v = \frac{\partial \omega}{\partial k} = \frac{\partial f}{\partial q}$ [4]) can only be applied locally.

As a first approximation, a weighted linear regression is used to estimate the wave velocity, i.e. the average slope of the dispersion relation $q \sim f(q)$. From the stability relation, we also know that *q*-modes with larger $\alpha(q)$ should contribute more to the wave velocity. Therefore we introduced a weighted factor $weight(q) = exp(-\frac{\alpha(q)^2}{2\sigma^2})$, $\sigma = max\,|\,\alpha(q)|$ to calculate the locally weighted linear regression [5]. Specifically, the least square method tries to minimize $w.\|Ab - Y\|^2 = \sum_{i=1}^{n} w(i)[f(i) - b_1 - b_2 q(i)]^2$, with data points $A = \begin{pmatrix} 1, q(1) \\ 1, q(2) \\ .. \\ 1, q(n) \end{pmatrix}$, $y = \begin{pmatrix} f(1) \\ f(2) \\ .. \\ f(n) \end{pmatrix}$, $w = \begin{pmatrix} w(1) \\ w(2) \\ .. \\ w(n) \end{pmatrix}$ to get the estimation $b = \begin{pmatrix} b_1 \\ b_2 \end{pmatrix}$. The overall average wave velocity is estimated by $v = |b_2|$. From the extrema, the least square gives $b = ((\sqrt{w}.A)^T(\sqrt{w}.A))^{-1}(\sqrt{w}.A)(\sqrt{w}.y)$, with the 'dot' being the Hadamard product (product by corresponding elements).

Supplementary Discussion: From the theoretical viewpoint, large waves are made up from dynamic modes with smaller spatial frequency q and nonzero temporal frequency $f(q) > 0$. Hence we can heuristically estimate the speed of large waves by the linear regression on these modes (i.e. q with $f(q) > 0$), and we found that it increases during the transition to wakefulness (Fig. 6B inset plot). Also, we can estimate the overall wave speed by a linear regression on all dynamic modes (i.e. all q); through doing this we observed a decrease in speed through this state transition (**main text Fig. 6b** inset).Thus, our neural mass model can further explain the increase in the speed of large waves (**main text Fig. 6b**, inset) [6] despite the decrease in the overall wave speed with the transition into wakefulness.

For both the awake and anesthetized states, the wave modes are those with small q and $f(q) > 0$. In this range of q, $\alpha(q)$ is large so these modes are more likely to emerge than those with a larger q. If only looking at this part, the linear wave theory predicts that the wave velocity is larger in the awake state, since the average slope of the dispersion relation $q \sim f(q)$ is larger (see **Supplementary Figs. 5b, c**). This explains that, in the experimental data, if we only focus on the large (global) waves (e.g. like in [6]), the wave speed is higher in the awake state (**main text Fig. 2c inset**). However, for the total group velocity (i.e. the overall velocity including both large and small waves), the observation is different. In the awake state (**Supplementary Fig. 5b**), Turing patterns (with larger q and $f(q) = 0$) are more likely to emerge, since $\alpha(q)$ is larger compared to the anesthetized state (**Supplementary Fig. 5c**). Thus,

from the viewpoint of the overall group velocity, the dispersion relation $q{\sim}f(q)$ should be corrected by the Turing patterns parts. Those Turing patterns have zero velocity so that the total wave velocity is lower in the awake state.

Long-range connection in the neural field model

Supplementary Discussion: We considered here the simple scenario of having only one weak long-range connection (with strength $\mu = 5$) in a noise-free system (**Supplementary Fig. 8**). We observed that activities propagate with a single wave front in the anaesthetized state (**Supplementary Fig. 8** Upper), whereas in the awake state, an ongoing wave may trigger the emergence of new waves at the long-range projection target location (**Supplementary Fig. 8** Lower). Even though the long-range projection strength is constant and similar in both states in our simulation, it is much more likely to trigger new waves when local neuronal excitability is high (as in the awake state; **Supplementary Fig. 8**).

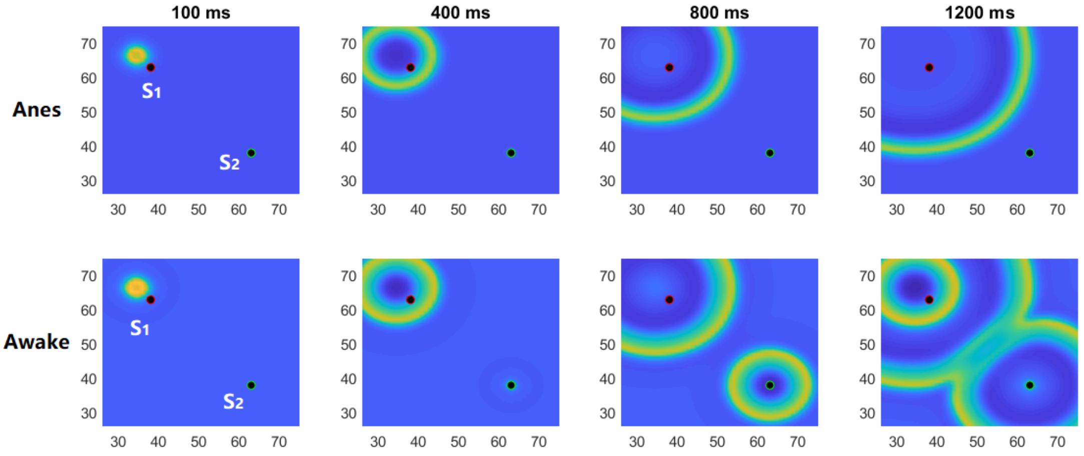

Supplementary Fig. 8: The effects of one long-range connection on wave patterns in anesthetized and awake states. Simulation of a long-range connection from site 1 to site 2, with a constant strength $\mu = 5$ in a noise-free system. In the awake state this long-range projection led to the induction of new wave while such effect is absent in the anesthetized state. Same color scale as Supplementary Fig. 6.

5. Selected pairs of cortical regions with different long-range connection strength

Supplementary Table 1. Regions with weak and strong long-range connection strength provided by Allen Mouse Brain atlas data[7]).

**Weak long-range connection**	from SSp-bfd to RSPagl	from Mos to SSp-n	from RSPd to SSp-bfd
**connection strength**	0	0	0
**Strong long-range connection**	from SSp-bfd to Mop	from Mos to SSp-tr	from RSPd to SSp-bfd

**connection strength**	2.677	1.237	1.514

Supplementary References

1. Rubino, D., Robbins, K. A. & Hatsopoulos, N. G. Propagating waves mediate information transfer in the motor cortex. *Nat. Neurosci.* **9**, 1549–1557 (2006).
2. Steyn-Ross, M. L., Steyn-Ross, D. A., Wilson, M. T. & Sleigh, J. W. Gap junctions mediate large-scale Turing structures in a mean-field cortex driven by subcortical noise. *Phys. Rev. E* **76**, 11916 (2007).
3. Yang, L., Dolnik, M., Zhabotinsky, A. M. & Epstein, I. R. Pattern formation arising from interactions between Turing and wave instabilities. *J. Chem. Phys.* **117**, 7259–7265 (2002).
4. Hayes, W. D. Group velocity and nonlinear dispersive wave propagation. *Proc. R. Soc. London. A. Math. Phys. Sci.* **332**, 199–221 (1973).
5. Cleveland, W. S. & Devlin, S. J. Locally weighted regression: an approach to regression analysis by local fitting. *J. Am. Stat. Assoc.* **83**, 596–610 (1988).
6. Shimaoka, D., Song, C. & Knöpfel, T. State-dependent modulation of slow wave motifs towards awakening. *Front. Cell. Neurosci.* **11**, 1–11 (2017).
7. Oh, S. W. *et al.* A mesoscale connectome of the mouse brain. *Nature* **508**, 207–214 (2014).
