# Supplementary material for: Complexity of cortical wave patterns of the wake mouse cortex
Source: Nat Commun. 2023 Mar 15;14:1434. doi: 10.1038/s41467-023-37088-6 (PMC10015011; doi:10.1038/s41467-023-37088-6)
Supplement: Supplementary file 7 — Description of Additional Supplementary Files [file 41467_2023_37088_MOESM7_ESM.pdf]

Description of Additional Supplementary Files

Supplementary Movie 1: Representative facial video clip from an anesthetized trial.

Supplementary Movie 2: Representative facial video clip from a post-woken trial.

Supplementary Movie 3: Representative facial video clip from a fully awake trial.
